# Chemical Motifs Associated with FAERS-Derived Severe Cutaneous Adverse Reaction Disproportionality Signals: An Interpretable Pharmacovigilance-Driven Cheminformatics Study

**DOI:** 10.3390/ijms27115062

**Published:** 2026-06-03

**Authors:** Yoshihiro Uesawa, Kaito Inden, Mizuho Asada

**Affiliations:** Department of Medical Molecular Informatics, Meiji Pharmaceutical University, Kiyose, Tokyo 204-8588, Japan

**Keywords:** SCARs, FAERS, disproportionality signal, pharmacovigilance-driven cheminformatics, signal classification, LightGBM, SHAP, chemical motifs, cluster-based validation, safety screening

## Abstract

Severe cutaneous adverse reactions (SCARs) are rare, life-threatening drug hypersensitivity syndromes. Although pharmacovigilance can identify drugs disproportionately reported with SCARs, it does not reveal which local chemistries recur among them. To address this, we assessed whether drugs with FAERS-derived SCAR disproportionality signals share interpretable chemical motifs. We screened FAERS data from 2004Q1 to 2024Q3, identified 5523 drugs with available Simplified Molecular-Input Line-Entry System (SMILES) representations, and constructed a signal-enriched dataset of 1676 compounds with nominally significant broad-SCAR associations after excluding predefined therapeutic/supportive confounders. Compounds were assigned to positive-signal [natural logarithm of reporting odds ratio (lnROR) > 0, *n* = 1219] or non-positive-signal (lnROR ≤ 0, *n* = 457) classes and encoded with 9753 explicitly mappable atom-centered local substructure descriptors. A LightGBM signal-classification model evaluated using random repeated nested cross-validation (six-fold outer × 50 repeats) achieved moderate internal discrimination (mean area under the receiver operating characteristic curve = 0.7041 ± 0.0337). Descriptor-space cluster-based repeated nested cross-validation, designed to reduce train–test structural leakage, yielded lower but still above-chance performance (mean ROC AUC = 0.6409; permutation *p* = 0.001), indicating that random-split estimates should be interpreted as optimistic for structurally novel compounds. Sensitivity analyses using minimum SCAR case-count thresholds and retention of predefined therapeutic/supportive drugs showed broadly similar performance and motif rankings. SHapley Additive exPlanations (SHAP) analysis revealed a fragment-level contrast: allylamine-like, ethanolamine-related, and diaminopropane-related motifs were associated with higher positive-signal class probability, whereas phenol and pyrimidine motifs were associated with lower positive-signal class probability. These findings suggest that FAERS-derived broad-SCAR signal direction is not chemically random within the selected dataset. Overall, the proposed framework should be viewed not as a direct predictor of absolute clinical SCAR risk but as an exploratory, pharmacovigilance-driven cheminformatics approach for prioritizing compounds and motif families for further SCAR-focused evaluation.

## 1. Introduction

### 1.1. Background: Severe Cutaneous Adverse Reactions

Severe cutaneous adverse reactions (SCARs) are rare, life-threatening drug hypersensitivity syndromes, including Stevens–Johnson syndrome (SJS), toxic epidermal necrolysis (TEN), drug reaction with eosinophilia and systemic symptoms (DRESS), and acute generalized exanthematous pustulosis (AGEP) [1]. These predominantly immune-mediated reactions extensively affect the skin and mucosa and can progress to systemic complications and death [2]. While SJS and TEN lie on a clinical severity spectrum defined by the extent of epidermal detachment and are associated with high mortality rates, DRESS and AGEP cause substantial acute morbidity and clinically important long-term sequelae [3,4,5].

The mechanisms underlying drug-induced SCARs are heterogeneous and unclear. According to the hapten hypothesis, reactive drug metabolites covalently modify self-proteins and generate neoantigens that trigger immune recognition [6]. Meanwhile, the pharmacological interaction (p–i) concept proposes that some drugs activate immune receptors through direct, noncovalent interactions without prior covalent binding [7]. In both frameworks, drug chemical structure is relevant as it influences bioactivation, covalent reactivity, or the molecular interactions preceding T-cell activation. Identifying structural patterns associated with SCAR-related signals therefore remains an important question at the intersection of pharmacovigilance, medicinal chemistry, and immunotoxicology.

### 1.2. Limitations of Current Approaches

Pharmacovigilance databases such as the FDA Adverse Event Reporting System (FAERS) support postmarketing signal detection by enabling analyses of drug–adverse event associations in extensive real-world reporting datasets [8]. However, FAERS-derived signals are based on disproportionality and do not directly quantify causal risk. Under-reporting, stimulated reporting, reporting volume, therapeutic context, comedication, disease indication, and patient-level factors can all influence these signals [9]. While pharmacovigilance databases effectively identify drugs that are disproportionately reported with SCAR-related events, they cannot independently explain which molecular features may underlie those associations.

So far, traditional structure–activity relationship (SAR) studies of SCARs have generally focused on individual drug classes. For instance, reactive metabolites of sulfonamide antimicrobials have long been implicated in hypersensitivity reactions [10], while aromatic anticonvulsants have been linked to SCAR risk in specific human leukocyte antigen (HLA) backgrounds [11]. These class-specific findings are mechanistically important; however, they do not provide a broad, drug-class-agnostic view of structure–signal relationships across therapeutic areas. Computational approaches that systematically analyze chemical structures across many compounds serve as a useful complement, particularly for the exploratory identification of candidate structural patterns rather than the establishment of causal determinants of risk.

### 1.3. Computational Approaches to SCARs

Classical quantitative SAR (QSAR) modeling systematically links molecular descriptors to experimentally grounded biological or toxicological endpoints [12]. In the context of adverse drug reactions (ADRs), QSAR-related models have been used to predict hepatotoxicity [13], cardiotoxicity [14], and skin sensitization [15]. The present study differs from such classical experimental-toxicity QSAR settings because its endpoint is a FAERS-derived disproportionality signal class. Therefore, we use the QSAR and cheminformatics literature as methodological context but frame the present analysis as pharmacovigilance-driven, interpretable structure–signal classification.

Nonetheless, several prior studies have demonstrated the applicability of in silico approaches to SCAR-related classification. For instance, Low et al. [16] combined molecular descriptors with pharmacovigilance-derived SJS labels from VigiBase and developed QSAR models to identify candidate structural alerts. Ambe et al. [17] applied deep neural networks to the Japanese Adverse Drug Event Report (JADER) database for broad SCAR prediction, demonstrating the utility of labels derived from spontaneous reports while prioritizing predictive performance over fragment-level interpretability. Li et al. [18] performed a comprehensive FAERS-based pharmacovigilance analysis of SCAR signals at the drug level; however, they did not implement a substructure-level, chemically interpretable structure–signal framework.

Together, these studies establish that labels derived from pharmacovigilance data can support computational analyses of SCARs; however, they differ in source database, endpoint scope, descriptor representation, modeling strategy, and structural interpretability. Consequently, pharmacovigilance-derived labels provide a pragmatic basis for exploratory structure–signal mining across diverse drug classes, provided that the resulting models are not interpreted as direct predictors of absolute clinical risk [19]. Notably, among descriptors, binary substructure fingerprints are particularly suited to interpretable structure–signal modeling because each bit can, in principle, be mapped to an identifiable chemical motif [20]. Predefined key sets such as Molecular ACCess System (MACCS) keys [21] enable direct interpretation within a fixed vocabulary but have limited granularity. By contrast, common fixed-length circular fingerprints such as extended-connectivity fingerprints (ECFPs) [22] provide high structural resolution but reduce descriptor-level traceability through hashing. Explicitly enumerated atom-centered local substructure descriptors circumvent this trade-off by preserving a one-to-one mapping between each descriptor and a canonical Simplified Molecular-Input Line-Entry System (SMILES) string, making them particularly suitable for fragment interpretation based on SHapley Additive exPlanations (SHAP). Meanwhile, among gradient-boosted decision tree algorithms, light gradient-boosting machine (LightGBM) has performed strongly in cheminformatics tasks involving high-dimensional sparse binary features [23]. When paired with SHAP, this model can provide both signal-classification discrimination and chemically interpretable feature attribution [24].

### 1.4. Objective

This study aimed to identify recurring chemical motifs in drugs with FAERS-derived SCAR disproportionality signals and to translate those motifs into interpretable safety hypotheses. Here, a “SCAR signal” refers to a drug-level disproportionality signal in the FAERS, meaning that broad SCARs are reported for a given drug more often than expected from the database’s background reporting distribution. Although this signal does not reflect incidence or establish causality, it serves as a practical surrogate for postmarketing concern about SCARs and can therefore support pharmacovigilance-driven, structure-based safety screening.

The specific research question was whether broad-SCAR-positive drugs in the FAERS share recognizable local chemistries that distinguish them from drugs on the opposite side of the same FAERS-based contrast. To address this question, we constructed an interpretable pharmacovigilance-driven signal-classification model using explicitly mappable local substructure descriptors and LightGBM. We then applied SHAP analysis to identify fragment motifs driving the separation between the two groups. The goal was not to estimate absolute clinical SCAR risk for arbitrary compounds but to extract chemically explicit clues that could support early safety assessment, mechanistic hypothesis generation, and prioritization of compounds for further SCAR-focused evaluation.

## 2. Results

### 2.1. Model Performance

A repeated nested cross-validation procedure (six-fold outer cross-validation repeated 50 times) yielded 300 outer-fold test evaluations. Table 1 summarizes the fold-level performance metrics across these 300 evaluations.

The model achieved a mean area under the receiver operating characteristic curve (ROC AUC) of 0.7041 ± 0.0337 and a mean precision–recall curve (PR AUC) of 0.8592 ± 0.0189. Its mean balanced accuracy was 0.6685 ± 0.0277, while its mean Matthews correlation coefficient (MCC) was 0.3167 ± 0.0509. Further, its sensitivity (SENS; 0.6984 ± 0.1151) was higher than its specificity (SPEC; 0.6386 ± 0.1190) on average, indicating that it was slightly more effective at identifying positive-signal compounds than at correctly classifying non-positive-signal compounds within the selected dataset. The corresponding row-normalized confusion matrix indicated that positive-signal compounds were classified as positive in 69.8% of outer-fold evaluations and non-positive-signal compounds were classified as non-positive in 63.9%. The larger fold-to-fold variation in sensitivity and specificity was consistent with class imbalance and endpoint-label heterogeneity.

Notably, the PR AUC should be interpreted relative to the positive-class prevalence of approximately 72.7% (1219/1676). A baseline classifier reflecting this prevalence would yield a PR AUC of approximately 0.73. The observed PR AUC of 0.8592 therefore indicates moderate improvement over this prevalence baseline, consistent with the ROC AUC results. Collectively, the above performance metrics indicate moderate internal ability to discriminate FAERS-derived SCAR signal direction within the selected, signal-enriched dataset, not clinically deployable prediction of absolute SCAR risk.

**Figure 1:**
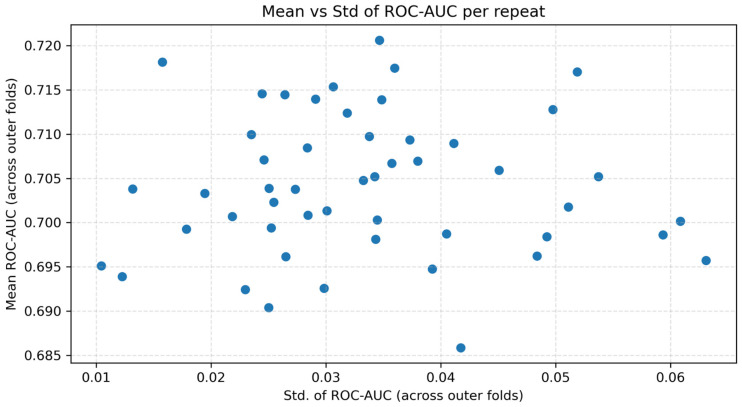
Stability of nested cross-validation performance across 50 repeated runs. Each point represents one repeat, with the mean ROC AUC across the six outer folds on the y-axis and the corresponding within-repeat standard deviation on the x-axis. The narrow clustering of points indicates that the internal random-split performance estimate was stable across repeated data partitions.

Figure 1 shows model-performance stability across the 50 repeated runs. Repeat-level mean ROC AUC ranged from 0.6859 to 0.7206 (overall mean = 0.7041, SD = 0.0080), and within-repeat standard deviation ranged from 0.0104 to 0.0631. Most repeats clustered within a relatively narrow band, indicating that the internal performance estimate was stable across repeated random data partitions. Notably, this stability supports the robustness of the internal random-split estimate but does not establish generalizability to external datasets or novel scaffolds.

### 2.2. Descriptor-Space Cluster-Based Validation

To assess the extent to which random-split performance might reflect structurally related compounds appearing in both training and test partitions, we performed descriptor-space cluster-based repeated nested cross-validation. Compounds were clustered as connected components of a Tanimoto/Jaccard similarity graph computed from the same binary Sub_* descriptor matrix used for modeling, using a threshold of 0.70. Whole clusters, rather than individual compounds, were then assigned to outer train or test folds.

This procedure produced 1105 descriptor-space clusters among 1676 compounds. The largest cluster contained 44 compounds, and 898 clusters were singletons. Across six outer folds repeated 50 times, all 300 outer evaluations had zero train–test cluster overlap. No test compound had a train-set nearest neighbor with Tanimoto similarity ≥ 0.70, and the maximum observed train–test Tanimoto similarity was 0.6993. By contrast, in the random-split comparison, 36,138 of 83,800 test-compound evaluations (43.1%) had a train-set nearest neighbor with Tanimoto similarity ≥ 0.70.

As expected, performance decreased under the more stringent validation scheme. Descriptor-space cluster-based repeated nested cross-validation yielded a mean ROC AUC of 0.6409, mean PR AUC of 0.8214, mean balanced accuracy of 0.6310, and mean MCC of 0.2415. A permutation test based on cluster-based out-of-fold predictions remained significant (OOF ROC AUC = 0.6576, null mean = 0.5005, null SD = 0.0157, *p* = 0.001). These results suggest that random-split performance should be interpreted as an optimistic internal estimate, while the cluster-based analysis supports more modest above-chance signal-classification ability under reduced structural leakage.

### 2.3. Sensitivity Analyses, Motif Stability, and Confusion-Matrix Summaries

Minimum case-count sensitivity analyses yielded performance close to the main random repeated nested cross-validation result. Using a minimum SCAR case-count threshold of ≥3 produced 1666 compounds and a mean ROC AUC of 0.7041, mean PR AUC of 0.8588, and mean MCC of 0.3186. Using a threshold of ≥5 produced 1654 compounds and a mean ROC AUC of 0.7012, mean PR AUC of 0.8565, and mean MCC of 0.3133. Retaining the predefined therapeutic/supportive drugs that were excluded from the main positive-signal set produced 1707 compounds and slightly lower performance (mean ROC AUC = 0.6903, mean PR AUC = 0.8554, mean MCC = 0.2947).

Motif-level stability was also examined across five analysis settings: the main random repeated nested cross-validation, descriptor-space cluster-based validation, minimum case count ≥3, minimum case count ≥5, and retention of predefined therapeutic/supportive drugs. Phenol (Sub_12841), ethanolamine (Sub_3431), and pyrimidine (Sub_5051) were retained among the top-ranked SHAP or split-importance features in all five settings. N-methyl-1,3-diaminopropane (Sub_10138) and the allylamine-like descriptor (Sub_106) were retained in four of five settings, whereas N-methylethanolamine (Sub_10148) was retained in two of five settings.

Confusion-matrix summaries were added to support classification-level interpretation. For the main random repeated nested cross-validation, the mean row-normalized sensitivity was 0.6984 and specificity was 0.6386. For the descriptor-space cluster-based validation, sensitivity was lower (0.5972) while specificity was slightly higher (0.6647). Because calibrated out-of-fold probability files were not retained uniformly for all sensitivity runs, calibration curves were not regenerated across analyses; instead, the saved outer-fold metrics were used to provide comparable confusion-matrix summaries.

### 2.4. Post Hoc Label-Randomization Check

A post hoc label-randomization check assessed whether the pooled out-of-fold prediction vector aligned with the observed labels more strongly than expected by chance. The pooled out-of-fold ROC AUC was 0.7177, compared with the mean fold-level ROC AUC of 0.7041 reported in Table 1. This difference emerges because the pooled statistic is derived from a single ROC curve constructed from all concatenated out-of-fold predictions, while the value listed in Table 1 is the arithmetic mean of 300 fold-specific ROC AUC values.

The null distribution from 1000 random label shuffles had a mean of 0.5013 and an SD of 0.0154, consistent with near-chance discrimination under random label assignment. The observed pooled out-of-fold ROC AUC exceeded all 1000 randomized values, yielding an empirical *p*-value of 0.001.

This result supports nonrandom alignment between the observed labels and pooled out-of-fold predictions. However, because randomization was applied to a fixed out-of-fold prediction vector and did not involve rerunning the full nested modeling pipeline for each permutation, the above result should be interpreted as a post hoc sanity check, not as a full pipeline-level permutation test.

### 2.5. Final Model for Interpretation

For the final interpretation model, hyperparameters were reoptimized on the full dataset, and the optimal number of boosting iterations was determined to be 116 by early stopping (internal cross-validated ROC AUC = 0.7244). Predicted probabilities were calibrated by Platt scaling with five-fold cross-validation, and a fixed classification threshold of 0.5 was applied. This refit model was used exclusively for feature-importance and SHAP analyses; the above repeated nested cross-validation results remain the primary internal estimate of discriminative performance.

### 2.6. Feature Importance

Table 2 lists the 10 descriptors with the highest LightGBM split count in the final interpretation model. Split count indicates how often a feature was used as a decision rule across the tree ensemble and therefore provides a simple measure of its utility for data partitioning.

Sub_12841 (phenol fragment; Oc1ccccc1) and Sub_5051 (pyrimidine fragment; c1cncnc1) were the most frequently employed features, each appearing in 16 splits. By contrast, Sub_106 (allylamine-like fragment; [C]C(=C)N) exhibited the highest total gain despite a lower split count, indicating that splits using this feature produced comparatively large improvements in the objective function.

**Figure 2:**
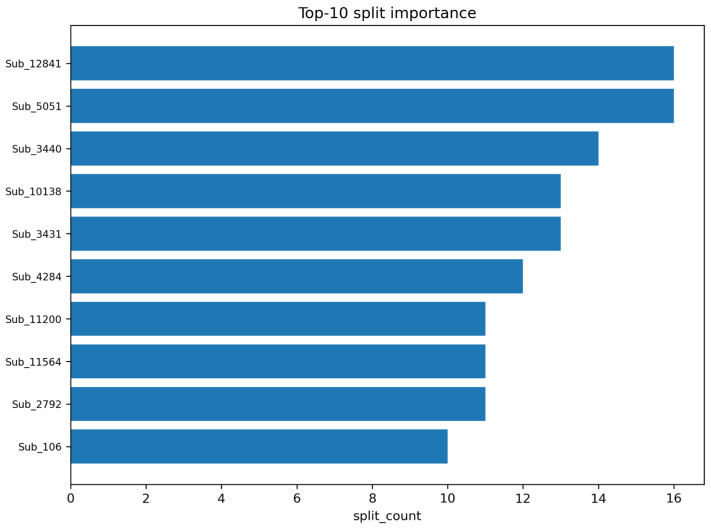
Top 10 substructure descriptors ranked by LightGBM split count in the final interpretation model. Bar length indicates how often each feature was selected as a splitting variable. See Table 2 and Supplementary Table S4 for descriptor annotations and SMILES mappings.

Descriptor annotations, class-level prevalence values, representative compounds, and exception examples are presented in Supplementary Table S3. Motif-retention stability across the main and sensitivity analyses is summarized in Supplementary Table S7. Figure 2 shows the top 10 features by split count.

### 2.7. SHAP Analysis

SHAP analysis was performed on the final interpretation model to quantify each descriptor’s contribution to individual predictions. Table 3 presents the top 10 features ranked by mean absolute SHAP value.

Among all features, Sub_12841 (phenol fragment) was the most influential, with the highest mean |SHAP| value (0.1727), followed by Sub_106 (allylamine-like fragment; 0.1031), Sub_3431 (ethanolamine fragment; 0.0846), and Sub_10138 (N-methyl-1,3-diaminopropane; 0.0832).

**Figure 3:**
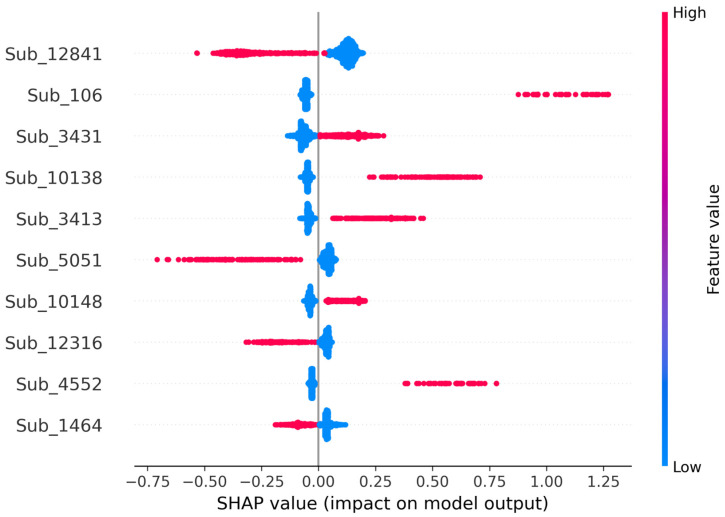
SHAP beeswarm plot of the top 10 substructure descriptors. Each dot represents one compound. The horizontal position indicates the SHAP value, and the color indicates feature state (pink, present; blue, absent). Features with right-shifted pink dots are associated with higher positive-signal class probability, whereas features with left-shifted pink dots are associated with lower positive-signal class probability within the selected dataset.

The SHAP beeswarm plot (Figure 3) indicated the direction of the above effects. Specifically, Sub_106 ([C]C(=C)N), Sub_3431 (NCCO), Sub_10138 (CNCCCN), Sub_4552 ([CH]OC(C)OC), and Sub_10148 (CNCCO) were generally associated with higher positive-signal class probability, whereas Sub_12841 (Oc1ccccc1), Sub_5051 (c1cncnc1), and Sub_1464 ([C]CCC[CH]) were associated with lower positive-signal class probability.

These directionality assignments should be interpreted at the motif-family level, not as a strict one-to-one mechanistic hierarchy, because the descriptor space contains nested and potentially correlated local fragments. Supplementary Table S3 lists representative and exception compounds containing the top-ranked motifs, and Supplementary Table S7 summarizes motif stability across five analysis settings. Phenol (Sub_12841), ethanolamine (Sub_3431), and pyrimidine (Sub_5051) were retained among the top-ranked SHAP or split-importance features in all five settings; N-methyl-1,3-diaminopropane (Sub_10138) and the allylamine-like descriptor (Sub_106) were retained in four of five settings. Notably, several descriptors, including Sub_106 and Sub_1464, are open-valence fragment patterns generated by local-environment enumeration and therefore should be interpreted as partial motifs rather than fully resolved standalone moieties.

#### Comparison of SHAP and Split-Importance Rankings

Five features appeared in the SHAP and split-importance top 10 lists: Sub_12841 (phenol fragment), Sub_5051 (pyrimidine fragment), Sub_10138 (N-methyl-1,3-diaminopropane), Sub_3431 (ethanolamine fragment), and Sub_106 (allylamine-like fragment). Both methods ranked Sub_12841 as the most important feature overall.

Concurrently, they also emphasized different aspects of model behavior. Notably, split count reflects how often a feature is used as a decision boundary in the tree ensemble, whereas the SHAP analysis reflects the magnitude and direction of its contribution to individual predictions. For example, Sub_3413 (glycinamide fragment) ranked highly in SHAP analysis but did not appear in the split-count top 10 list, suggesting meaningful local effects despite less frequent usage for data partitioning. Conversely, several features ranked highly in split-count analysis but not in SHAP analysis, indicating frequent use for tree partitioning without correspondingly large per-sample effect sizes. This complementarity supports both interpretation modes for motif-family-level model interpretation.

### 2.8. Chemical Structures of Key Substructures

**Figure 4:**
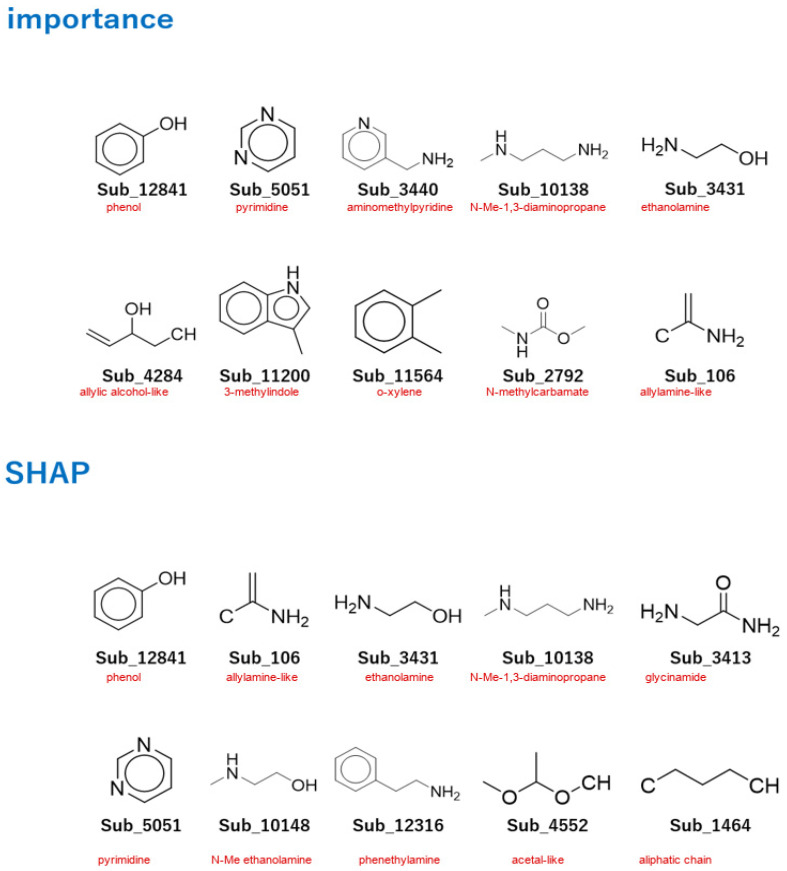
Chemical structures of top-ranked substructure descriptors from LightGBM split importance and SHAP analysis, annotated with Sub_ IDs and motif-family labels. Upper panel: Top 10 descriptors by LightGBM split count (Table 2). Lower panel: Top 10 descriptors by mean absolute SHAP value (Table 3). Within each panel, descriptors descend in rank from left to right and then from top to bottom. The structures were drawn from canonical fragment SMILES documented in the feature–SMILES mapping file (Supplementary Table S4). Descriptors appearing in both panels indicate concordance between split-based feature usage and SHAP-based contribution magnitude. Because several descriptors represent fragment-like or open-valence local patterns extracted by atom-centered local substructure enumeration, the drawings should be interpreted as partial structural motifs, not fully resolved standalone chemical moieties. This applies particularly to Sub_106, Sub_1464, and Sub_4552.

Figure 4 shows the chemical structures corresponding to the top-ranked descriptors identified by LightGBM split importance and SHAP analysis, with added motif-family labels to aid interpretation. Structure assignments were derived from the feature–SMILES mapping file (Supplementary Table S4). Notably, because several descriptors represent fragment-like local environments with open valences, the structures should be interpreted as partial motifs, not as fully resolved standalone chemical entities.

## 3. Discussion

### 3.1. Model Performance in Context

The key finding of this study is that broad-SCAR concern detected in the FAERS is not chemically random within the selected signal-enriched dataset. With explicitly interpretable local substructure descriptors, the model achieved stable internal discrimination under random repeated nested cross-validation (mean ROC AUC = 0.7041 ± 0.0337) and retained above-chance signal-classification ability under descriptor-space cluster-based repeated nested cross-validation (mean ROC AUC = 0.6409). Most importantly, allylamine-like, ethanolamine-related, and diaminopropane-related motifs characterized the positive-signal side of the contrast, whereas phenol and pyrimidine motifs characterized the non-positive-signal side.

Overall, the primary contribution is not simply classifier training but the translation of a pharmacovigilance-derived contrast into a chemically interpretable motif pattern. In practical terms, this analysis moves beyond listing SCAR-associated drugs and asks a more useful exploratory question for medicinal chemistry and safety science: what types of local chemistry repeatedly characterize drugs that generate postmarketing SCAR concern in spontaneous-report data?

Several sources of biological and reporting heterogeneity are nevertheless likely to remain beyond the scope of a structure-only model. These include pharmacological class composition, co-medication patterns, disease indication, exposure prevalence, reporting behavior, host immune status, and genetic susceptibility factors such as HLA background [25]. Accordingly, the model should be understood not as an attempt to fully explain SCAR pathogenesis or as a bedside risk-prediction tool but as a structure-guided framework for identifying compounds and motif families whose local chemistry warrants closer mechanistic or pharmacovigilance follow-up.

#### 3.1.1. Position Relative to Previous Studies

Prior computational studies have demonstrated that pharmacovigilance-derived labels can support in silico analysis of SCAR-related outcomes. For instance, Low et al. [16] established this concept for SJS using VigiBase-derived labels and classical cheminformatics approaches. Ambe et al. [17] extended this concept to broad SCAR using JADER and deep learning, whereas Li et al. [18] provided a detailed FAERS-based pharmacovigilance characterization of SCAR at the drug level without fragment-level structural interpretation.

This study contributes a fragment-resolved FAERS-based perspective by linking the direction of broad-SCAR disproportionality to chemically traceable local motifs. Its value therefore lies less in classical experimental-toxicity QSAR prediction or predictive-performance benchmarking than in a chemically explicit, hypothesis-generating framework for interpreting FAERS-derived SCAR concern.

#### 3.1.2. Descriptor Choice and Validation Design

The adopted descriptor representation was selected primarily for chemical traceability and interpretability. Because each retained descriptor mapped directly to a canonical fragment SMILES string, model-important features could be interpreted as recognizable local motifs rather than anonymous hashed bits. This property is particularly valuable for SHAP-based interpretation and for linking model behavior to plausible medicinal-chemistry and immunotoxicology hypotheses.

Notably, this study was not designed to benchmark alternative descriptor families or model classes. No direct comparison with MACCS, ECFPs, physicochemical descriptors, or simpler classifiers was performed. This choice reflected the primary focus on chemical interpretability through direct descriptor-to-SMILES traceability rather than predictive benchmarking. Whether the motif associations identified here remain stable across alternative descriptor and algorithm choices is an important question for future research. The random repeated nested cross-validation design yielded an internally stable estimate under repeated random repartitioning of the selected dataset. Because random splitting may place structurally related compounds in both training and test partitions, we added descriptor-space cluster-based repeated nested cross-validation as a more stringent sensitivity validation. The performance decrease under cluster-based splitting indicates that the random-split estimate should be regarded as an optimistic internal estimate rather than evidence of scaffold-level generalizability.

### 3.2. Structural Motifs Associated with Disproportionate SCAR Reporting

On the positive-signal side, the dominant pattern was not a single isolated fragment but a coherent motif family centered on allylamine-like, ethanolamine-related, and diaminopropane-related substructures. This suggests that drugs appearing as broad-SCAR-positive in the FAERS are enriched in local chemistries that may be compatible with bioactivation, reactive-intermediate formation, or immune-relevant amine functionality. In other words, the model did not simply reflect individual drug identities; it extracted a recurring statistical motif pattern shared across multiple positive-signal compounds.

Among these examined motifs, the allylamine-like fragment (Sub_106; [C]C(=C)N) had the greatest SHAP magnitude. One possible interpretation is that allylamine-related local chemistry may be compatible with metabolic activation pathways that generate reactive intermediates capable of covalent protein modification. Such a mechanism is conceptually consistent with the hapten hypothesis of drug hypersensitivity, wherein drug-derived reactive species contribute to neoantigen formation and downstream T-cell activation [6,26,27]. However, the current data do not establish fragment-level causality, and the observed association may also reflect pharmacological class composition or reporting structure within the FAERS.

The ethanolamine (Sub_3431; NCCO), N-methyl-1,3-diaminopropane (Sub_10138; CNCCCN), and N-methylethanolamine (Sub_10148; CNCCO) motifs similarly indicate an amino-alcohol/diamine-rich motif family associated with higher positive-signal class probability. These functional groups may facilitate oxidative metabolism, reactive-intermediate formation, or immune-relevant interactions, including drug-mediated alteration of HLA-peptide repertoires [28]. Supplementary Table S3 lists representative compounds containing these motifs. These examples ground the motif family in recognizable drugs but should not be interpreted as proof of fragment-level causality.

The acetal-like fragment (Sub_4552; [CH]OC(C)OC) also had a positive-direction association. This observation is mechanistically difficult to interpret and warrants particular caution because the descriptor is a local open-valence motif rather than a complete functional-group assignment. Overall, the positive-direction features are best interpreted as candidate marker motifs associated with disproportionate SCAR reporting within the selected FAERS-derived dataset.

### 3.3. Structural Motifs Associated with the Opposite Side of the SCAR Contrast

Another important contribution of this study is that the model identified a clear counter pattern. Phenol was the strongest overall feature and, together with pyrimidine, consistently represented the opposite side of the FAERS-based SCAR contrast. This pattern is not merely a negative finding; it indicates that the model captured a chemically interpretable separation: one motif family characterized drugs with disproportionate SCAR reporting, whereas another characterized the comparator group.

The phenol result warrants special emphasis because phenol was the top SHAP feature in the entire model. At minimum, this indicates that phenol-containing local chemistry is a strong discriminator of the non-positive side of the present FAERS contrast. This observation should not be interpreted as evidence that phenol-containing drugs are intrinsically protective against SCARs. Within the selected dataset, the phenol motif instead served as a strong marker of the non-positive-signal side. Supplementary Table S3 lists representative compounds bearing this motif, and their distribution should be considered when interpreting this result. Several nonmutually exclusive explanations may apply. First, the phenol finding may partly reflect therapeutic-class composition in the selected dataset. Second, inverse or non-positive disproportionality in spontaneous-report data can also be conditioned by exposure prevalence, indication-specific reporting, comedication patterns, and differential use across patient groups, allowing a chemically common fragment to appear on the non-positive side without implying biological protection. Third, phenolic chemistry may affect redox or reactive-intermediate behavior under some conditions [29]; however, the current observational design cannot isolate intrinsic chemical effects from dataset composition or reporting structure. Accordingly, the phenol finding is best interpreted as a data-driven negative-direction marker within the present FAERS-derived contrast, not as evidence of a protective effect.

The pyrimidine fragment (Sub_5051; c1cncnc1) warrants the same conservative interpretation. Its association with lower positive-signal class probability may reflect reduced susceptibility to specific bioactivation pathways, therapeutic-class composition, or both. Supplementary Table S3 lists representative compounds containing this motif. This study therefore identifies pyrimidine as part of the non-positive side of the chemical contrast but does not assign it a causal or protective role.

### 3.4. Comparison of SHAP and Split Importance

Comparing SHAP and split-count rankings clarified complementary aspects of model behavior. Notably, split count measures how often a feature is used to partition the feature space, whereas the SHAP value captures the magnitude and direction of its contribution to individual predictions. In tree-based models, SHAP/TreeSHAP provides local and global feature-attribution summaries for ensemble predictions [30], and explainable artificial intelligence is increasingly used in drug discovery to connect model behavior to chemically interpretable hypotheses [31]. The observed agreement between the two rankings, particularly for phenol-, pyrimidine-, ethanolamine-, diaminopropane-, and allylamine-like motifs, strengthens the interpretation that these motif families were central to the fitted model.

Meanwhile, their discrepancies also provide insights. Features that ranked highly by SHAP analysis but not by split count may have exerted strong local effects in specific regions of the descriptor space, whereas features ranked highly by split count but not by SHAP analysis may have served as frequent but lower-impact partitioning rules. However, because the descriptor space contains correlated and nested fragments, neither ranking should be overinterpreted at the level of a single isolated fragment. The most defensible interpretation is instead at the motif-family level.

### 3.5. Limitations

Several limitations of this study warrant acknowledgment, particularly because the endpoint is a selected FAERS-derived signal class rather than an experimentally measured toxicity outcome.

*FAERS-derived label limitation.* Endpoint labels were derived from spontaneous-report pharmacovigilance data and therefore inherit known sources of bias, including under-reporting, stimulated reporting, comedication effects, confounding by indication, therapeutic pathway effects, and differential exposure prevalence [9]. The labels represent disproportionality-based associations in the FAERS rather than adjudicated causal SCAR risk.

*Selection-dependent labeling and multiple testing.* Only compounds with nominally significant drug-level associations (*p* < 0.05) were retained, while no multiple-testing correction was applied during screening. This choice was made to construct a sensitivity-oriented, signal-enriched subset for exploratory structure–signal analysis, but it also implies that some retained labels may reflect false positives or selection-dependent noise. The resulting classes should therefore be understood as analytical labels linked to the selected FAERS-derived dataset, not as definitive biological categories.

*Analytical contrast-group limitation.* The adopted non-positive-signal class is an analytical contrast group defined by reporting direction rather than a validated low-risk reference class. Accordingly, differences between the two classes should be interpreted as differences within a signal-enriched FAERS subset, not as contrasts between hazardous and clinically safe drugs.

*Validation limitation.* This study now includes descriptor-space cluster-based repeated nested cross-validation as a sensitivity validation, but it still does not include fully external validation. The cluster-based analysis reduced train–test structural similarity by assigning whole Tanimoto/Jaccard descriptor clusters to either training or test folds, and performance decreased from a mean ROC AUC of 0.7041 under random repeated nested cross-validation to 0.6409 under cluster-based validation. This decrease supports the interpretation that the random-split estimate is optimistic for structurally novel compounds. Similarly, the motif associations identified by SHAP analysis should be treated as dataset-specific hypotheses pending external validation [32].

*Label-randomization limitation.* The statistical check reported for the random-split model in Section 2.4 was performed by shuffling labels against a fixed pooled out-of-fold prediction vector. The check therefore supports nonrandom alignment between observed labels and pooled predictions but should not be interpreted as a full pipeline-level permutation test. For the cluster-based validation, a corresponding out-of-fold permutation test remained significant (*p =* 0.001).

*Descriptor and interpretation limitations.* The binary local-fragment descriptors encode only two-dimensional topological information and do not capture full three-dimensional geometry, stereochemical detail, or explicit host–drug interaction context. Furthermore, the descriptor set contains nested and correlated motifs, and several top-ranked features are open-valence fragment patterns rather than fully specified functional groups. In such high-dimensional correlated descriptor spaces, SHAP attribution can be shared or redistributed among related fragments. The results are therefore most appropriately interpreted as model-specific statistical summaries at the motif-family level, not as unique causal explanations for isolated fragments.

*Endpoint aggregation.* SJS, TEN, DRESS, and AGEP were aggregated into a single broad-SCAR endpoint. Although this broad endpoint increases sample size and supports exploratory analysis, it may obscure subtype-specific structure–signal relationships.

*Asymmetric confounder exclusion and therapeutic-class composition.* The predefined exclusion of 31 therapeutic/supportive confounders was intentionally applied only when a compound showed a nominally significant positive association with the SCAR endpoint. This step reduced plausible treatment-related false positives but altered class composition asymmetrically by design. A sensitivity analysis retaining these predefined therapeutic/supportive drugs yielded slightly lower but broadly similar performance (mean ROC AUC = 0.6903; mean MCC = 0.2947), suggesting that the main result was not solely driven by this exclusion rule. Nevertheless, because the modeling table did not include curated ATC or therapeutic-class annotations, some motif associations may still reflect therapeutic-class composition, indication, or treatment-context effects rather than independent fragment-level effects.

*Preprocessing and reproducibility limitations.* Although a concise summary of the upstream FAERS preprocessing workflow and molecular standardization procedure is presented in the Supplementary Methods, the reproducibility of pharmacovigilance-derived structure–signal analyses remains sensitive to upstream choices such as name normalization, ingredient mapping, reporter-role filtering, deduplication, and structure standardization.

### 3.6. Future Directions

Several directions could reinforce the present framework. First, future studies should evaluate alternative label constructions, including multiple-testing-controlled labels and continuous or variance-weighted formulations based on the natural logarithm of the reporting odds ratio (lnROR) and its uncertainty. In this revision, minimum case-count sensitivity analyses produced results close to the main analysis, but a full continuous lnROR model would require a different uncertainty structure and weighting scheme. Second, fully external validation will help determine whether the identified motif families generalize beyond the present FAERS-derived dataset. Third, direct benchmarking against alternative descriptor families and baseline models would clarify the trade-off between predictive performance and chemical interpretability. Finally, integrating chemical descriptors with pharmacogenomic factors, particularly HLA genotype, and developing subtype-specific models for SJS/TEN, DRESS, and AGEP may provide a more mechanistically resolved overview of SCAR-associated structure–signal relationships.

## 4. Materials and Methods

### 4.1. Data Source and Endpoint Definition

Drug–adverse reaction association data were extracted from the FAERS for the first quarter of 2004 through the third quarter of 2024 (2004Q1–2024Q3). SCARs were defined using 21 preferred terms (PTs) from the Standardised MedDRA Query (SMQ) for severe cutaneous adverse reactions [33] in the Medical Dictionary for Regulatory Activities (MedDRA). The 21 PTs were acute generalised exanthematous pustulosis, AGEP–DRESS overlap, bullous haemorrhagic dermatosis, cutaneous vasculitis, dermatitis bullous, dermatitis exfoliative, dermatitis exfoliative generalised, drug reaction with eosinophilia and systemic symptoms, epidermal necrosis, erythema multiforme, erythrodermic atopic dermatitis, exfoliative rash, generalised bullous fixed drug eruption, oculomucocutaneous syndrome, severe cutaneous adverse reaction, SJS–TEN overlap, skin necrosis, Stevens–Johnson syndrome, target skin lesion, toxic epidermal necrolysis, and toxic skin eruption. These PTs were used exactly as defined by the MedDRA SMQ, without manual modification. This broad SMQ-based definition was adopted to maximize sensitivity in pharmacovigilance screening and therefore encompassed heterogeneous SCAR-related phenotypes rather than a single clinically homogeneous entity.

From the FAERS, 5523 unique drug substances with obtainable SMILES structural representations were initially identified. The SMILES representations were retrieved primarily from PubChem using normalized active-ingredient names as query identifiers, and the structures were standardized to canonical non-isomeric SMILES, as described in the Supplementary Methods. Disproportionality analysis was performed at the PRIMARYID level. Duplicate PRIMARYID–drug and PRIMARYID–adverse-event records were collapsed before constructing the binary drug-presence and event-presence matrices, respectively. A report was classified as SCAR-positive if it contained at least one of the 21 constituent SCAR PTs. For each drug substance, a 2 × 2 contingency table was constructed across PRIMARYIDs, and the ROR, its natural logarithm (lnROR), and 95% confidence intervals were derived from this table [34]. Two-sided Fisher’s exact test *p*-values were calculated using the raw contingency tables. For ROR and confidence-interval calculations, continuity correction was applied uniformly to all contingency tables by adding 0.5 to all four cells.

Broader upstream FAERS preprocessing steps, including drug-name normalization, active-ingredient mapping, reporter-role filtering, and duplicate handling at the case/version level, were executed before the current modeling workflow. A concise summary of these preprocessing procedures is presented in the Supplementary Methods to improve reproducibility.

To suppress potential false-positive associations arising from therapeutic or supportive co-reporting, drug substances potentially associated with SCAR treatment, supportive care, or complication management and showing a statistically significant positive association with the SCAR endpoint (lnROR > 0 and Fisher’s exact test *p* < 0.05) were excluded from a predefined list. These excluded agents amounted to 31 and included glucocorticoid receptor agonists, immunosuppressants, a vasodilator, and antiviral agents. Clinical guidelines, systematic reviews, and case-series evidence supported the exclusion rationale; Supplementary Table S1 provides the full drug-level list with evidence sources. This exclusion step was intentionally asymmetric and applied only to compounds showing nominally significant positive associations with the SCAR endpoint.

After therapeutic confounders were excluded, only compounds with a statistically significant association with the SCAR endpoint (Fisher’s exact test *p* < 0.05) were retained for modeling, while compounds with *p* ≥ 0.05 were excluded. Each retained drug substance was then assigned a binary label based on the direction of its FAERS-derived association: compounds with lnROR > 0 and *p* < 0.05 were labeled positive-signal, whereas those with lnROR ≤ 0 and *p* < 0.05 were labeled non-positive-signal. This procedure yielded a final dataset of 1676 compounds, including 1219 positive-signal and 457 non-positive-signal compounds.

Notably, this labeling scheme captures the direction of FAERS-derived disproportionality among statistically significant compounds and should not be interpreted as a direct estimate of absolute SCAR liability. The non-positive-signal class comprised compounds with a statistically significant inverse or non-positive association in the FAERS (lnROR ≤ 0, *p* < 0.05). Such inverse associations may arise from differential exposure patterns, indication-specific reporting, co-medication structure, or less frequent use in populations at high SCAR risk and should not be equated with intrinsic clinical safety. The non-positive-signal class therefore served as an analytical contrast group defined by reporting direction within the selected dataset, not as a validated low-risk reference class. Because compounds with *p* ≥ 0.05 were excluded, the resulting dataset was a signal-enriched subset rather than a comprehensive sample of all marketed drug substances. No multiple-testing correction was applied during drug screening. This choice prioritized sensitivity in constructing a signal-rich dataset for exploratory structure–signal analysis; accordingly, the resulting labels should be interpreted as selection-dependent analytical labels, not confirmatory drug-level pharmacovigilance signals.

### 4.2. Molecular Descriptors

Chemical structures were represented by binary substructure descriptors generated by an in-house program (Substructure_Descriptor v6; unpublished) implemented with the RDKit cheminformatics library [35]. For each molecule, atom-centered local environments were enumerated at radii of 1–4 bonds with RDKit’s FindAtomEnvironmentOfRadiusN function. Each environment was converted to a submolecule with PathToSubmol and expressed as a canonical non-isomeric SMILES string. The union of all unique fragment SMILES observed in the dataset defined the descriptor dictionary. Each compound was then encoded as a binary vector indicating the presence or absence of each fragment. After zero-frequency columns were removed, 9753 descriptors were retained for model development. Original feature identifiers were preserved for traceability; consequently, the retained descriptors had non-consecutive original IDs rather than sequentially renumbered IDs.

This descriptor representation was selected primarily for chemical traceability and interpretability. Compared with predefined key sets such as the MACCS [21], the dataset-adaptive descriptor space captured a broader range of motifs present in the studied chemical space. Compared with common fixed-length circular fingerprints such as ECFPs [22], each descriptor mapped one-to-one onto a canonical SMILES string, avoiding bit-collision ambiguity and enabling direct mapping from model-important features to identifiable chemical motifs. Notably, the present study was not designed to benchmark alternative fingerprint families; rather, the descriptor choice was made to support chemically explicit interpretation of model-important motifs.

### 4.3. Model Development

#### 4.3.1. Algorithm

LightGBM (version 4.6.0), an efficient implementation of gradient-boosted decision trees, was used as the classification algorithm [23]. This model family was selected because it handles sparse, high-dimensional binary features and supports downstream SHAP-based interpretation. Early stopping with 100-round patience was used during training to reduce overfitting.

#### 4.3.2. Hyperparameter Optimization

Hyperparameters were optimized with Optuna [36], a Bayesian optimization framework. In each inner cross-validation cycle, 25 parameter configurations (trials) were sampled and evaluated. The hyperparameter search space is summarized in Table 4.

#### 4.3.3. Class Imbalance Handling

The final modeling dataset contained 1219 positive-signal and 457 non-positive-signal compounds. In LightGBM, scale_pos_weight scales the loss contribution of positive-class samples. Setting this parameter to 0.375 (457/1219) reduced the loss contribution of the majority positive-signal class and balanced loss weighting between the two classes during training.

#### 4.3.4. Probability Calibration

Predicted probabilities were calibrated with Platt scaling (sigmoid method) [37]. During nested cross-validation, calibration was performed using four-fold internal cross-validation on each outer-training set. Meanwhile, for the final model, five-fold cross-validation calibration was applied. Notably, the four-fold cross-validation used for Platt-scaling calibration was independent of the six-fold inner cross-validation used for Optuna-based hyperparameter selection; however, both procedures were restricted to the outer-training partition and did not involve the held-out outer-test fold.

### 4.4. Model Validation

#### 4.4.1. Repeated Nested Cross-Validation

Model performance was assessed using a repeated nested cross-validation design [38]. The outer loop comprised six-fold cross-validation repeated 50 times, producing 300 outer-fold test evaluations, while the inner loop used six-fold cross-validation for hyperparameter selection with Optuna. This nested design was intended to provide an internal estimate of discriminative performance while minimizing information leakage between model selection and evaluation on the outer test folds. Because random splits rather than scaffold-based splits were used, structurally related compounds may have appeared in both the training and test partitions. Therefore, the resulting performance estimates should be interpreted as internal estimates from random splits, not as evidence of scaffold-level generalizability.

#### 4.4.2. Descriptor-Space Cluster-Based Repeated Nested Cross-Validation

To evaluate performance under reduced structural leakage, we additionally performed descriptor-space cluster-based repeated nested cross-validation. Pairwise compound similarity was computed as Tanimoto/Jaccard similarity on the binary Sub_* descriptor matrix. A graph was constructed by connecting compound pairs with similarity ≥0.70, and connected components of this graph were treated as descriptor-space clusters. Group-aware repeated stratified splitting was then used so that all compounds from a given cluster were assigned to either the training partition or the test partition in each outer fold.

The cluster-based validation used the same six outer folds, 50 repeats, LightGBM search space, and evaluation metrics as the random repeated nested cross-validation. Within the cluster-based scheme, hyperparameter optimization used five-fold group-aware inner cross-validation. After fold construction, train–test cluster overlap and nearest-neighbor train–test Tanimoto similarity were computed for each outer evaluation to verify split integrity. A random-split nearest-neighbor Tanimoto comparison was also generated to contextualize the degree of structural overlap expected under random partitioning.

#### 4.4.3. Sensitivity Analyses

Three additional sensitivity analyses were performed. First, the model was rerun after requiring at least three SCAR reports per retained compound. Second, the model was rerun after requiring at least five SCAR reports per retained compound. Third, the predefined therapeutic/supportive drugs excluded from the main positive-signal set were retained to evaluate the influence of the asymmetric confounder-exclusion rule. For each sensitivity setting, repeated nested cross-validation metrics, permutation-test summaries where available, and motif-retention patterns were compared with the main analysis.

#### 4.4.4. Evaluation Metrics

For each outer fold, the following metrics were computed: ROC AUC, PR AUC, accuracy (ACC), balanced accuracy (BA), F1 score, MCC, SENS, and SPEC. Summary statistics, including the mean, standard deviation, minimum, maximum, and median, were computed for all outer-fold test evaluations. Fold-level summary statistics are reported herein as the primary performance estimates. Row-normalized confusion-matrix summaries were derived from saved sensitivity and specificity metrics to support classification-level interpretability. Separately, all out-of-fold predictions were concatenated into a single vector, and a pooled out-of-fold ROC AUC was also computed. This pooled statistic is reported separately where relevant and differs numerically from the arithmetic mean of the fold-level ROC AUC values.

#### 4.4.5. Post Hoc Label-Randomization Check

The post hoc label-randomization check assessed whether the pooled out-of-fold prediction vector aligned with the observed labels more strongly than expected by chance. The out-of-fold predictions from the nested cross-validation pipeline were held fixed, class labels were randomly shuffled 1000 times, and ROC AUC was recalculated for each shuffle to generate a null distribution. The resulting empirical *p*-value should therefore be interpreted as a sanity check of alignment between the fixed out-of-fold predictions and observed labels, not as a full pipeline-level permutation test.

#### 4.4.6. Final-Model Construction

For model interpretation, a final model was constructed by rerunning Optuna-based hyperparameter optimization on the complete dataset and determining the number of boosting iterations with early stopping. Predicted probabilities were calibrated by Platt scaling with five-fold cross-validation, and a fixed classification threshold of 0.5 was applied. The final-model hyperparameters are reported in Supplementary Table S5. This refit model was used only for interpretation and feature ranking; the repeated nested cross-validation results remain the primary internal performance estimates.

### 4.5. Model Interpretation

#### 4.5.1. SHAP Analysis

SHAP values were computed for the final interpretation model to quantify each descriptor’s contribution to individual predictions. Features were ranked by mean absolute SHAP value (mean |SHAP|) across all samples. Because the descriptor space contained nested and potentially correlated local fragments, SHAP rankings were interpreted at the motif-family level rather than as a strict one-to-one mechanistic hierarchy.

#### 4.5.2. LightGBM Feature Importance

Beyond SHAP analysis, LightGBM’s built-in feature-importance measures were extracted from the final model. Split count and total gain were recorded. The top 10 features ranked by split count were documented to complement the SHAP-based ranking.

#### 4.5.3. Substructure Mapping

Each binary descriptor was mapped to its corresponding chemical substructure based on the feature–SMILES mapping file. Chemical drawings of the top-ranked descriptors were prepared for visual interpretation. For fragment-like descriptors with open valences, the resulting drawings were treated as partial local motifs, not fully resolved standalone chemical entities.

### 4.6. Software, Data Availability, and Reproducibility

All analyses were performed in Python 3.9.23 (conda-forge) on Windows 10. The major packages used were LightGBM 4.6.0, (https://github.com/microsoft/LightGBM, accessed on 9 April 2026), scikit-learn 1.6.1 (https://scikit-learn.org, accessed on 9 April 2026), Optuna 4.4.0 (https://optuna.org, accessed on 9 April 2026), SHAP 0.48.0 (https://github.com/shap/shap, accessed on 9 April 2026), NumPy 2.0.2 (https://numpy.org, accessed on 9 April 2026), pandas 2.3.1 (https://pandas.pydata.org, accessed on 9 April 2026), and matplotlib 3.9.4 (https://matplotlib.org, accessed on 9 April 2026). A fixed random seed of 32 was used for all stochastic procedures. To improve reproducibility, a concise summary of the upstream FAERS preprocessing workflow and molecular standardization procedure is presented in the Supplementary Methods, including drug-name normalization, active-ingredient mapping, reporter-role filtering, deduplication rules, and structure-standardization steps. Supplementary Table S1 lists the excluded therapeutic/supportive confounders with rationale and evidence sources. Supplementary Table S2 lists the included compounds with class labels, lnROR, 95% confidence intervals, nominal *p*-values, and standardized SMILES. Supplementary Table S3 lists top-ranked features with class-level prevalence, representative compounds, and exception examples. Supplementary Table S4 provides the feature–SMILES mapping. Supplementary Table S5 reports the final model hyperparameters and training configuration. Supplementary Tables S6–S8 provide the cluster-based validation, sensitivity analysis, motif stability, confusion matrix, and calibration availability summaries added in response to reviewer comments.

## 5. Conclusions

This study addressed a practical exploratory question: do drugs with FAERS-derived disproportionate SCAR reporting share recurring chemical motifs? The answer was yes within the selected signal-enriched dataset. An interpretable pharmacovigilance-driven cheminformatics model constructed from explicitly traceable local substructure descriptors revealed a distinct fragment-level contrast: allylamine-like, ethanolamine-related, and diaminopropane-related motifs characterized the positive-signal side, whereas phenol and pyrimidine motifs characterized the non-positive-signal side.

These findings suggest that FAERS-derived SCAR signal direction is not chemically featureless and that fragment-resolved cheminformatics can extract structurally meaningful hypotheses from pharmacovigilance data. The present framework should therefore be viewed not as a direct predictor of absolute clinical SCAR risk but as an interpretable, structure-guided safety-screening approach for identifying compounds and motif families that warrant closer SCAR-focused evaluation. The reduced but significant performance observed under descriptor-space cluster-based validation further supports a cautious interpretation: the model captures signal beyond simple random-split memorization, while remaining exploratory and dataset-dependent.

## Figures and Tables

**Table 1:** Summary of nested cross-validation performance across 300 outer-fold test evaluations. ROC AUC: Area under the receiver operating characteristic curve; PR AUC: area under the precision–recall curve; ACC: accuracy; BA: balanced accuracy; F1: F1 score; MCC: Matthews correlation coefficient; SENS: sensitivity; SPEC: specificity.

Metric	Mean	SD	Min	Max	Median
ROC AUC	0.7041	0.0337	0.5872	0.7971	0.7063
PR AUC	0.8592	0.0189	0.7910	0.9038	0.8599
ACC	0.6821	0.0569	0.4194	0.7957	0.6882
BA	0.6685	0.0277	0.5840	0.7691	0.6690
F1	0.7561	0.0701	0.3415	0.8619	0.7662
MCC	0.3167	0.0509	0.1853	0.5140	0.3198
SENS	0.6984	0.1151	0.2069	0.9261	0.7094
SPEC	0.6386	0.1190	0.3026	0.9868	0.6406

**Table 2:** Top 10 features by light gradient-boosting machine (LightGBM, version 4.6.0) split count in the final interpretation model.

Rank	Feature	SMILES	Substructure Description	Split Count	Gain
1	Sub_12841	Oc1ccccc1	Phenol fragment	16	63.5992
2	Sub_5051	c1cncnc1	Pyrimidine fragment	16	45.1674
3	Sub_3440	NCc1cccnc1	Aminomethylpyridine fragment	14	30.8971
4	Sub_10138	CNCCCN	N-Methyl-1,3-diaminopropane	13	28.9546
5	Sub_3431	NCCO	Ethanolamine fragment	13	23.5982
6	Sub_4284	[CH]CC(O)C=C	Allylic alcohol-like fragment	12	34.8441
7	Sub_11200	Cc1c[nH]c2ccccc12	3-Methylindole fragment	11	26.0074
8	Sub_11564	Cc1ccccc1C	o-Xylene fragment	11	25.9274
9	Sub_2792	CNC(=O)OC	N-Methylcarbamate fragment	11	23.5489
10	Sub_106	[C]C(=C)N	Allylamine-like fragment	10	105.4242

**Table 3:** Top 10 features ranked by mean absolute SHAP value in the final interpretation model.

Rank	Feature	SMILES	Substructure Description	Mean |SHAP|
1	Sub_12841	Oc1ccccc1	Phenol fragment	0.1727
2	Sub_106	[C]C(=C)N	Allylamine-like fragment	0.1031
3	Sub_3431	NCCO	Ethanolamine fragment	0.0846
4	Sub_10138	CNCCCN	N-Methyl-1,3-diaminopropane	0.0832
5	Sub_3413	NCC(N)=O	Glycinamide fragment	0.0754
6	Sub_5051	c1cncnc1	Pyrimidine fragment	0.0693
7	Sub_10148	CNCCO	N-Methylethanolamine fragment	0.0574
8	Sub_12316	NCCc1ccccc1	Phenethylamine fragment	0.0564
9	Sub_4552	[CH]OC(C)OC	Acetal-like fragment	0.0528
10	Sub_1464	[C]CCC[CH]	Aliphatic chain fragment	0.0519

**Table 4:** Hyperparameter search space for LightGBM optimization with Optuna.

Hyperparameter	Type	Range/Values
num_leaves	Categorical	{8, 16, 32, 64, 128}
max_depth	Categorical	{−1, 4, 6, 8, 12}
learning_rate	Categorical	{0.01, 0.05, 0.1}
min_child_samples	Categorical	{5, 10, 20, 30, 40, 60, 80}
subsample	Continuous	[0.5, 1.0]
subsample_freq	Categorical	{0, 1, 5}
colsample_bytree	Continuous	[0.4, 1.0]
reg_alpha	Continuous	[1 × 10^−8^, 10.0] (log scale)
reg_lambda	Continuous	[1 × 10^−8^, 10.0] (log scale)
min_split_gain	Categorical	{0.0, 0.01, 0.05, 0.1}

## Data Availability

The data presented in this study were derived from a resource available in the public domain: the U.S. FDA Adverse Event Reporting System (FAERS), available at https://fis.fda.gov/extensions/FPD-QDE-FAERS/FPD-QDE-FAERS.html (accessed on 9 April 2026). The processed datasets and descriptor–SMILES mapping metadata supporting the conclusions of this article are provided in the Supplementary Materials. The analysis code will be made available by the authors on request.

## Supplementary Materials

The following supporting information can be downloaded at https://www.mdpi.com/article/10.3390/ijms27115062/s1.

## Abbreviations

The following abbreviations are used in this manuscript:

ADR	adverse drug reactions
AGEP	acute generalized exanthematous pustulosis
BA	balanced accuracy
DRESS	drug reaction with eosinophilia and systemic symptoms
FAERS	FDA Adverse Event Reporting System
HLA	human leukocyte antigen
JADER	Japanese Adverse Drug Event Report
LightGBM	light gradient-boosting machine
MCC	Matthews correlation coefficient
PT	preferred terms
SCAR	severe cutaneous adverse reactions
SMILES	Simplified Molecular-Input Line-Entry System
SMQ	Standardised MedDRA Query
TEN	toxic epidermal necrolysis
