# Supplementary material for: Chemical Motifs Associated with FAERS-Derived Severe Cutaneous Adverse Reaction Disproportionality Signals: An Interpretable Pharmacovigilance-Driven Cheminformatics Study
Source: Int J Mol Sci. 2026 Jun 3;27(11):5062. doi: 10.3390/ijms27115062 (PMC13257069; doi:10.3390/ijms27115062)
Supplement: Supplementary file 1 [file ijms-27-05062-s001.zip › ijms-4324442_supplementary_methods.docx]

Supplementary Methods

Manuscript: Chemical Motifs Associated with FAERS-Derived Severe Cutaneous Adverse Reaction Disproportionality Signals: An Interpretable Pharmacovigilance-Driven Cheminformatics Study

Authors: Yoshihiro Uesawa*, Kaito Inden and Mizuho Asada

This Supplementary Methods document expands on the Materials and Methods section of the main manuscript by providing operational details relevant to reproducibility. Preprocessing steps that were performed upstream of the final modeling pipeline are described at the procedural level, and remaining transparency limitations are acknowledged in the main manuscript.

# S1. FAERS data acquisition

## S1.1. Source database and time window

Drug–adverse-event association data were extracted from the U.S. Food and Drug Administration Adverse Event Reporting System (FAERS) public release, covering reports submitted between 2004 Q1 and 2024 Q3, inclusive. The FAERS quarterly tables used in preprocessing were DEMO (demographics), DRUG (drug entries), REAC (adverse reactions), and RPSR (report source).

## S1.2. Endpoint definition (broad SCAR)

Severe cutaneous adverse reactions (SCAR) were defined as a single composite endpoint covering 21 MedDRA Preferred Terms (PTs) drawn from the Standardised MedDRA Query (SMQ) for severe cutaneous adverse reactions. The 21 PTs are listed in Section 2.1 of the main manuscript and were not manually modified during preprocessing. A report was classified as SCAR-positive if at least one of these PTs appeared in the REAC table for that PRIMARYID. This broad-SCAR endpoint was selected to maximize sensitivity for pharmacovigilance screening and therefore covers heterogeneous SCAR-related phenotypes rather than one clinically homogeneous condition.

# S2. Drug-name normalization and active-ingredient mapping

The FAERS DRUG table contains free-text drug entries that are heterogeneous in granularity, including proprietary names, generic names, multi-component combinations, dosage-form descriptors, abbreviations, and misspellings. Active-ingredient mapping was applied before disproportionality analysis so that drug entries could be summarized at the active-ingredient level.

**String normalization.** All FAERS drug strings were converted to uppercase; leading and trailing whitespace was removed; common dosage-form suffixes (e.g., TABLETS, INJECTION, CREAM), salt designators (e.g., HCL, SODIUM, CITRATE), and hyphen or slash separators were standardized or removed; and punctuation variants were normalized before dictionary matching.

**Synonym resolution.** Normalized strings were matched against an internal active-ingredient dictionary derived from RxNorm, the WHO Anatomical Therapeutic Chemical (ATC) classification, and PubChem synonym lists, with manual curation of FAERS-prevalent ambiguous strings.

**Combination-product handling.** Drug entries representing fixed-dose combinations were split into their individual active ingredients before mapping, and each component was treated as a separate drug record for that PRIMARYID.

**Generic-class terms.** Generic FAERS class strings, such as STEROIDS without further specification, were retained as separate analytical units rather than being assigned to a single member of the class; one such generic term (“Steroids”) appears in Supplementary Table S1.

Drug entries that could not be mapped to a defined active ingredient under the above rules were excluded from the disproportionality analysis. The complete upstream mapping dictionary is not enumerated within the present report; this limitation is acknowledged in Section 4.5 of the main manuscript.

# S3. PRIMARYID-level duplicate handling

The unit of analysis for disproportionality estimation was the PRIMARYID, the FAERS field identifying an individual safety report. Within the PRIMARYID-level pipeline used for the present study, the following duplicate-collapsing rules were applied.

**Drug-presence matrix.** Multiple DRUG-table rows for the same PRIMARYID and the same mapped active ingredient were collapsed to a single drug-presence indicator before construction of the 2 × 2 contingency table. This collapsing was applied regardless of drug-role code.

**Event-presence matrix.** Multiple REAC-table rows for the same PRIMARYID and the same Preferred Term were similarly collapsed to a single event-presence indicator before the SCAR-positive report flag was evaluated.

**CASEID/version-level handling.** FAERS reports can be amended over time, generating multiple PRIMARYIDs that share a CASEID and represent successive versions of the same underlying case. CASEID/version-level deduplication, such as retaining only the most recent version per CASEID, was not applied within the analytical pipeline used for the modeled dataset. The analysis therefore operates at the PRIMARYID level, and the potential impact of follow-up versions is acknowledged as a limitation.

The PRIMARYID-level duplicate-collapsing rules above were applied uniformly to all 5,523 drug substances initially identified before the disproportionality screen and the therapeutic/supportive confounder exclusion described in Section S6.

# S4. Reporter-role and drug-role considerations

The FAERS RPSR and DRUG tables encode the role of the reporter and the role of each drug within a report. In the present analysis, these fields were handled as follows.

**Reporter occupation.** No restriction by reporter occupation (e.g., physician, pharmacist, consumer, or other reporter type) was applied at the PRIMARYID level.

**Drug role.** All drug-role codes, including primary suspect, secondary suspect, concomitant, and interacting, were treated as drug presence within a PRIMARYID for the purpose of constructing the binary drug-presence matrix. This deliberately inclusive definition was intended to capture FAERS-reported drug-event associations broadly rather than to restrict the analysis to suspected causality assigned by the reporter.

**Country of report and report type.** No restriction by country of origin or by FAERS report type (initial vs. follow-up, expedited vs. periodic) was applied; the analysis used the union of all reports available in the 2004 Q1–2024 Q3 window.

These choices were fixed before the disproportionality screen and are documented here for transparency. Their impact on the resulting disproportionality estimates was not separately evaluated in the present study.

# S5. Disproportionality estimation

For each mapped active ingredient i, a 2 × 2 contingency table was constructed at the PRIMARYID level over the broad-SCAR endpoint:

|  | **SCAR** | **non-SCAR** |
| --- | --- | --- |
| **Drug i present** | a | b |
| **Drug i absent** | c | d |

The reporting odds ratio (ROR), its natural logarithm (lnROR), and the 95% confidence interval for the ROR were computed using the standard analytical expression with the Haldane–Anscombe continuity correction. A value of 0.5 was added to all four cells of every contingency table for lnROR and confidence-interval calculation.

lnROR = ln[((a + 0.5)(d + 0.5)) / ((b + 0.5)(c + 0.5))]

SE(lnROR) = sqrt[1/(a + 0.5) + 1/(b + 0.5) + 1/(c + 0.5) + 1/(d + 0.5)]

95% CI(lnROR) = lnROR ± 1.96 × SE(lnROR)

95% CI(ROR) = exp[95% CI(lnROR)]

The continuity correction (+0.5 to all four cells) was applied uniformly to every drug for the ROR and confidence-interval calculation; this guarantees that lnROR and its confidence interval are finite even when one or more cells equal zero.

Two-sided Fisher’s exact test p-values were computed from the raw, uncorrected 2 × 2 tables. The continuity correction was applied only to the ROR and confidence intervals; the Fisher p-values were not affected by the +0.5 adjustment. Drug-level lnROR, ROR, 95% confidence intervals, nominal Fisher’s exact test p-values, and standardized SMILES for the 1,676 retained compounds are reported in Supplementary Table S2.

# S6. Therapeutic/supportive confounder exclusion

To reduce false-positive signals arising from drugs co-reported in FAERS as part of SCAR treatment, supportive care, or complication management rather than as suspected causative agents, an evidence-based exclusion list was applied before the p < 0.05 retention rule.

The exclusion rule was operationalized as follows:

The exclusion list consisted of 31 entries (Supplementary Table S1) curated a priori from clinical guidelines, international consensus statements, systematic reviews, and case-series evidence on SCAR management. The list comprises 25 glucocorticoid receptor agonists, one generic FAERS class string (“Steroids”), two immunosuppressive or antineoplastic agents used in SCAR-related management contexts, two antiviral nucleoside analogues used to treat HHV-6/CMV reactivation in DRESS/DIHS, and one haemorrheologic xanthine derivative reported in supportive microcirculatory care of TEN.

Each candidate active ingredient on the exclusion list was excluded from the modeling dataset only if it satisfied both predefined exclusion criteria at the time of screening: (i) lnROR > 0 against the broad-SCAR endpoint, and (ii) two-sided Fisher’s exact test p < 0.05 against the broad-SCAR endpoint.

Drugs on the exclusion list that did not meet both criteria were retained in the modeling dataset. The criteria were evaluated drug by drug; no drug was excluded purely on the basis of class membership.

Evidence sources for each excluded drug are provided in Supplementary Table S1. For selected excluded drugs, drug-level disproportionality statistics were not retained in the exported table; however, all excluded agents satisfied the predefined exclusion criteria at the time of screening.

After the therapeutic/supportive confounder exclusion and the p < 0.05 retention rule were both applied, the modeling dataset comprised 1,676 compounds: 1,219 in the positive-signal class and 457 in the non-positive-signal class. All compounds in the modeling dataset had p < 0.05 against the broad-SCAR endpoint.

# S7. Compound-level chemical-structure retrieval and standardization

## S7.1. PubChem SMILES retrieval

For each retained active ingredient, a chemical structure was retrieved from PubChem according to the following protocol.

The active-ingredient string used as the query was the same string passed to the disproportionality pipeline after normalization and synonym resolution (Section S2).

Lookups were performed against the PubChem Compound (CID) database using the active-ingredient name, with InChI and CAS-number cross-references where available.

When a PubChem query returned multiple candidate CIDs, the candidate with the smallest CID whose record corresponded to a parent compound (free-base or unsalted neutral form, where applicable) was retained. CIDs corresponding to salts, hydrates, isotope-labeled variants, deuterated derivatives, or stereoisomeric mixtures were deprioritized unless the parent compound could not be uniquely identified.

When a PubChem query returned no chemically interpretable parent compound, the substance was not included in the modeling dataset. The main manuscript reports 5,523 drug substances for which SMILES representations could be obtained; substances without retrievable SMILES were not part of those 5,523 candidates.

## S7.2. RDKit standardization

Each retrieved SMILES was processed with RDKit using the following standardization steps.

**Parsing.** The SMILES string was parsed with Chem.MolFromSmiles using standard RDKit sanitization.

**Salt/mixture stripping.** If the parsed molecule contained more than one disconnected fragment, such as a multi-component salt or co-crystal, the largest organic fragment by heavy-atom count was retained and the remaining fragments were discarded.

**Charge handling.** No automated neutralization or protonation-state adjustment was applied. Formal charges present in the PubChem record were retained in the parsed molecular graph.

**Tautomer handling.** No automatic tautomer canonicalization was applied; the canonical SMILES emitted by RDKit therefore reflects the tautomeric form present in the PubChem record.

**Stereochemistry.** Stereochemical annotations (@, @@, /, \) present in the source SMILES were retained where present in the PubChem record. The descriptor program operates on the molecular graph and extracts radius-defined local environments independently of whether stereochemical annotations are retained in the compound-level string.

**Canonicalization.** The parsed and standardized molecule was re-emitted as a canonical SMILES using Chem.MolToSmiles(canonical=True). The canonical SMILES values reported in Supplementary Table S2 are the strings produced by this step.

## S7.3. Compound-level vs. fragment-level SMILES

**Compound-level SMILES (Supplementary Table S2).** These are RDKit-canonical SMILES retaining stereochemical annotations where present in the source PubChem record. They may therefore contain notation such as [C@H] or /C=C/.

**Fragment-level SMILES (Supplementary Table S4).** These are canonical fragment SMILES emitted by the descriptor program (Substructure_Descriptor v6) for radius-defined atom-centered local environments. Fragment SMILES may contain bracketed open-valence atoms such as [C], [CH], [CH2], or [N] to denote atoms whose full valence is not represented in the extracted local environment.

This distinction allows descriptor-level chemical traceability while retaining a fragment-resolved feature space for modeling and interpretation.

# S8. Substructure descriptor generation and filtering

The substructure descriptor procedure followed the steps described in Section 2.2 of the main manuscript with the following operational details.

The descriptor program (Substructure_Descriptor v6; Y. Uesawa, 2025) was invoked in radius mode with a radius range of 1–4 bonds.

For each compound, every heavy atom was used as a center, and the local atomic environment within the specified radius was extracted using Chem.FindAtomEnvironmentOfRadiusN.

The local environment, defined as a set of bonds within the specified radius around the center atom, was converted to a submolecule using Chem.PathToSubmol and re-emitted as a canonical SMILES string.

A pre-filtered descriptor dictionary comprising 16,529 enumerated descriptor IDs was generated during substructure enumeration. After restricting the descriptor matrix to the final 1,676-compound modeling dataset and removing columns with zero total frequency, 9,753 binary substructure descriptors were retained for model development. Because original descriptor IDs were preserved for traceability, the retained feature IDs are non-consecutive.

Each compound was encoded as a binary indicator vector in which 1 indicated that the fragment was present in the compound and 0 indicated that it was absent. Fragment count information was not retained in this mode.

The descriptor program does not explicitly retain the generating radius (1–4) for each fragment after canonicalization. Supplementary Table S4 therefore reports a heuristic atom-count proxy for the radius (≤2 atoms, approximately r = 1; 3–5 atoms, approximately r = 2; 6–8 atoms, approximately r = 3; ≥9 atoms, approximately r = 4). This proxy is used only for annotation and was not used in model training.

# S9. LightGBM training, calibration, and validation

The LightGBM training pipeline is described in Sections 2.3–2.4 of the main manuscript. The Optuna search space, final selected hyperparameters, boosting/training settings, calibration configuration, repeated nested-cross-validation configuration, post hoc label-randomization check, random seed, and package versions are reported in Supplementary Table S5.

**scale_pos_weight derivation.** The scale_pos_weight parameter was set to the ratio of non-positive-signal to positive-signal compounds in the modeling dataset: 457/1219 ≈ 0.375. This value was held fixed at 0.375 across all outer folds, inner folds, Optuna trials, and the final refit model.

**Probability calibration scope.** Platt scaling (sigmoid method) was applied inside each outer training set during nested cross-validation using 4-fold internal cross-validation. For the final refit model used in SHAP analysis, a separate 5-fold calibration procedure was applied. Calibration was applied to predicted probabilities; threshold-based metrics used a fixed threshold of 0.5.

**Post hoc label-randomization check.** The pooled out-of-fold predictions obtained from the repeated nested cross-validation pipeline were held fixed. Class labels were randomly shuffled 1,000 times using NumPy random-number generation initialized from the fixed seed of 32, and ROC AUC was recalculated for each shuffled label vector. The null distribution had a mean of 0.5013 and an SD of 0.0154. The observed pooled out-of-fold ROC AUC was 0.7177 and exceeded all 1,000 randomized values, yielding an empirical p-value of 0.001. Because the model was not retrained for each randomization, this procedure is described as a post hoc label-randomization check rather than as a full pipeline-level permutation test.

# S10. Software environment and reproducibility

All analyses were performed under a fixed software environment: Windows 10; Python 3.9.23 from the conda-forge channel; NumPy 2.0.2; pandas 2.3.1; scikit-learn 1.6.1; LightGBM 4.6.0; Optuna 4.4.0; SHAP 0.48.0; matplotlib 3.9.4; and tqdm 4.67.1. A single random seed of 32 was propagated to NumPy, LightGBM, the Optuna sampler, and all cross-validation splitters.

# S11. Summary of the data flow

The end-to-end data flow can be summarized as follows.

• FAERS quarterly extracts (2004 Q1–2024 Q3) → drug-name normalization and active-ingredient mapping (Section S2).

• PRIMARYID-level duplicate handling of drug-presence and event-presence matrices (Section S3).

• PRIMARYID-level 2 × 2 contingency table per active ingredient → ROR with 95% CI (continuity correction +0.5 applied to all cells) and two-sided Fisher’s exact test p-value from raw uncorrected counts (Section S5).

• 5,523 active ingredients initially identified for which a PubChem SMILES was retrievable.

• PubChem retrieval and RDKit standardization (Section S7).

• Predefined therapeutic/supportive confounder exclusion (n = 31; Section S6 and Supplementary Table S1).

• Significance-based retention (Fisher’s p < 0.05) → 1,676 compounds in the modeling dataset (1,219 positive-signal, 457 non-positive-signal; Supplementary Table S2).

• Radius-defined atom-centered local substructure descriptor generation (Section S8) → 9,753 retained descriptors after filtering (Supplementary Table S4).

• LightGBM training with repeated nested cross-validation (6 × 50 = 300 outer evaluations) and a 1,000-randomization post hoc label-randomization check (Section S9 and Supplementary Table S5).

• Final-model refit on the full dataset for SHAP and feature-importance analysis (Supplementary Tables S3 and S5).

# S12. Reviewer-requested validation and sensitivity analyses

Additional analyses were performed to address reviewer requests concerning structural similarity across random splits, motif robustness, classification-level interpretation, and sensitivity to case-count and therapeutic/supportive-confounder handling.

## S12.1. Descriptor-space cluster-based validation

Compounds were clustered using the same binary Sub_* descriptor matrix used for model training. Pairwise Tanimoto/Jaccard similarity was computed on the binary descriptor vectors, and connected components were defined at a similarity threshold of 0.70. Outer train/test assignment was then performed at the whole-cluster level rather than at the individual-compound level. This validation scheme generated 1,105 descriptor-space clusters among 1,676 compounds, with a maximum cluster size of 44 and 898 singleton clusters. Across 300 outer evaluations, train and test sets had no cluster overlap, and the maximum train-test nearest-neighbor Tanimoto similarity was 0.6993. Cluster-based performance metrics, fold-level cluster-integrity summaries, the comparison with random splits, and the cluster-based out-of-fold label-randomization check are reported in Supplementary Table S6.

## S12.2. Sensitivity analyses and motif stability

Sensitivity analyses repeated the modeling pipeline after applying alternative minimum SCAR case-count thresholds and after retaining the predefined therapeutic/supportive drug list that had been excluded from the primary analysis. Motif stability was evaluated by tracking whether selected motif families appeared among the top-ranked descriptors by SHAP magnitude or split-based importance across the main and sensitivity analyses. Sensitivity summaries, raw outer-fold metrics, and motif-stability matrices are reported in Supplementary Table S7.

## S12.3. Confusion matrices and calibration availability

Row-normalized confusion matrices were reconstructed from the saved sensitivity and specificity metrics for each outer fold. For the cluster-based validation analysis, pooled confusion counts were reconstructed from the saved fold-level class counts and saved sensitivity/specificity metrics. Calibration curves were not regenerated for the submitted runs because the out-of-fold y_score values required for uniform calibration-curve construction were not retained for all analyses. The confusion-matrix summaries and calibration-availability note are reported in Supplementary Table S8.

# S13. Supplementary table file

The accompanying Excel workbook ijms-4324442_supplementary_tables_S1-S8.xlsx contains Supplementary Tables S1-S8. The first worksheet provides an index linking each supplementary item to the corresponding worksheet(s).
